# A Community Health Worker–Based Program for Elderly People With Hypertension in Indonesia: A Qualitative Study, 2013

**DOI:** 10.5888/pcd12.140530

**Published:** 2015-10-15

**Authors:** Riana Rahmawati, Beata Bajorek

**Affiliations:** Author Affiliations: Beata Bajorek, Graduate School of Health, School of Pharmacy, University of Technology Sydney, Australia, and Department of Pharmacy, Royal North Shore Hospital, Sydney, Australia. Dr Rahmawati is also affiliated with Pharmacology Department, Islamic University of Indonesia, Sleman, Yogyakarta, Indonesia.

## Abstract

**Introduction:**

Hypertension is prevalent in the elderly, but treatment is often inadequate, particularly in developing countries. The objective of this study was to explore the role of a community-based program in supporting patients with hypertension in an Indonesian rural community.

**Methods:**

A qualitative study comprising observation and in-depth interviews was conducted in an Integrated Health Service Post for the Elderly (IHSP-Elderly) program in Bantul district (Yogyakarta province). Eleven members of IHSP-Elderly program (ie, hypertensive patients), 3 community health workers (CHWs), and 1 district health staff member were interviewed to obtain their views about the role of the IHSP-Elderly program in hypertension management. Data were analyzed using thematic analysis.

**Results:**

CHWs played a prominent role as the gatekeepers of health care in the rural community. In supporting hypertension management, CHWs served members of the IHSP-Elderly program by facilitating blood pressure checks and physical exercise and providing health education. Members reported various benefits, such as a healthier feeling overall, peer support, and access to affordable health care. Members felt that IHSP-Elderly program could do more to provide routine blood pressure screening and improve the process of referral to other health care services.

**Conclusion:**

CHWs have the potential to liaise between rural communities and the wider health care system. Their role needs to be strengthened through targeted organizational support that aims to improve delivery of, and referral to, care. Further study is needed to identify the key factors for effective CHW-based programs in rural communities and the incorporation of these programs into the health care system.

## Introduction

Hypertension, a risk factor for cardiovascular disease, stroke, and premature death, is prevalent in the elderly (1). However, treatment of hypertension in the elderly is often inadequate, particularly in developing countries, where only 10% of cases are controlled (2). In Indonesia, a developing country in Southeast Asia, the prevalence of hypertension among those aged 65 or older is 60%, making it the most common chronic disease (3).

One key strategy to help scale up hypertension management is to involve the community; patient empowerment is the cornerstone of primary health care and chronic disease management (4). Programs involving community health workers (CHWs) in the United States have increased the proportion of patients with controlled blood pressure (5,6). The role of CHWs in facilitating access to health care services for patients with particular diseases has been studied in some developing countries (7). For example, a study in Uganda described the role of CHWs in supporting the management of pneumonia and malaria (8). Few studies describe CHWs’ role managing hypertension in developing countries (9).

In Indonesia, the Integrated Health Service Post for the Elderly (IHSP-Elderly), a national CHW-based program, has been promoting healthy aging since 1997; the program focuses on screening for and managing chronic diseases in low socioeconomic population subgroups (10). This study explored how the IHSP-Elderly programs could play a role in improving hypertension management for patients in rural communities in the Bantul district, Yogyakarta. In this district, hypertension is one of the most common diseases treated in primary health care facilities (11). The perspectives of CHWs, members of the IHSP-Elderly program (ie, patients), and local government health policy advisors were examined.

## Methods

### Study setting and design

This qualitative study took place in a village in the rural Bantul district of Yogyakarta province in Indonesia (Box) in March 2013. Yogyakarta province (population, 3.5 million) is in Java, the country’s most populous island; almost 52% of 238 million Indonesians live there (14). The life expectancy in Yogyakarta province is 74.1 years (15).

**Box. Bantul District, Yogyakarta Province, Indonesia**

Population, 930,276 (12)

Elderly population (aged ≥60 y), 110,729 (12)

Economy based on subsistence agriculture (12)

Muslim majority (91%) (12)

861 of 933 subvillages have an IHSP-Elderly program (13)

Sociocultural climate related to the implementation of IHSP-Elderly program:

• Both men and women are members

• Most (90%) community health workers are women (13)

• Freedom of conversation between sexes is accepted

• Freedom of expression is accepted (ie, people can freely exchange their views)

• Music and physical exercise in the IHSP-Elderly program are welcomed by members (12)

• Home visitation by CHWs can be undertaken without security concerns

This study used observation and in-depth face-to-face interviews to explore perspectives on the role of the IHSP-Elderly program in the management of hypertension. A semistructured approach used a purpose-designed interview guide (Table 1), developed after reviewing the relevant literature (16–18). A separate set of questions was used for each group of participants. The questions were pilot tested for clarity and relevance. The validity of findings was confirmed through independent analysis of data by the researchers and through respondent validation.

**Table 1:** Interview Guide for Participants, Study of the Role of a Community-Based Program for Elderly People With Hypertension, Indonesia, 2013

Questions for members of Integrated Health Service Post (IHSP) for the Elderly
1. How long have you had hypertension (since early diagnosis)?
2. What kind of symptoms do you have relating to hypertension?
3. What kind of medication have you taken?
4. What is your opinion about IHSP-Elderly activities?
5. Why do you think the activities of the IHSP-Elderly are useful?
6. What do you think about blood pressure examination by CHWs in the IHSP-Elderly?
7. What is your opinion about the role of CHWs in the IHSP-Elderly to support hypertension treatment?
8. How can the IHSP-Elderly improve its service in hypertension management?
**Questions for community health workers**
1. What kind of activities does the IHSP-Elderly organize for healthy aging?
2. Since you are a volunteer, what do you expect to be actively involved in the IHSP-Elderly?
3. What do you usually do after diagnosing members with high blood pressure?
4. How do you get the necessary information and skills relating to hypertension?
5. What are the barriers to implementing the hypertension program?
6. What are your suggestions for future programs?
**Questions for district health staff member**
1. Why is the IHSP-Elderly program important for communities in the district?
2. What has the government done to support the IHSP-Elderly program?
3. What are the expectations of the district health office for the IHSP-Elderly in regard to the hypertension program?

All participants were informed about the study via a purpose-designed information sheet and were required to provide written consent before participation. For 3 participants who were unable to read fluently, a researcher provided a comprehensive verbal explanation of study requirements before obtaining their consent. All participants were literate enough to write their name and provide a written signature. No participants received a financial reward for their interview. The study was approved by the Bantul district health office responsible for authorizing the conduct of research studies. The study protocol and other supporting documents were reviewed. Recruitment of participants began after approval was obtained to ensure no conflict of interest.

### Recruitment of participants

Nationally, there are 69,500 IHSP-Elderly programs (19). Our study’s sampling frame comprised 861 IHSP-Elderly programs in 27 community health centers in the Bantul District. First, an online random number generator was used to select 1 community health center and then 1 IHSP-Elderly program. In the village selected for participation, the elderly population consisted of 117 men and women; the local IHSP-Elderly program provides care for up to 70 members, and it has 3 CHWs. 

The study had 3 types of participants: members of the IHSP-Elderly program (patients), CHWs serving the IHSP-Elderly program, and a district health staff member. Patients were purposely sampled according to the following inclusion criteria: aged 60 years or older, active in the IHSP-Elderly program for at least 1 year, and with diagnosed hypertension (confirmed by IHSP-Elderly record, systolic blood pressure ≥140 mm Hg and ≥diastolic blood pressure 90 mm Hg). The appropriate sample size for members was initially determined to be 10 (20,21), but we recruited an additional patient because theme saturation was not reached until we interviewed 11 members. All 3 CHWs serving the IHSP-Elderly program were invited to participate in the study and participated. These CHWs were local residents who were trained to serve the IHSP-Elderly program, and each had done so for at least 3 years. The head of the district health office designated the staff member in charge of the IHSP-Elderly program as the participant representing the district health office.

### Data collection and analysis

A typical IHSP-Elderly event (a weekly meeting) was observed to collect descriptive information on activities and interactions (verbal and nonverbal behavior) among members and CHWs. Each member pays 1,000 Indonesian rupiah (IDR) (<USD $0.10) to attend a meeting. In comparison, the standard patient fee for a consultation in the community health center is 9,000 IDR (22) and about 50,000 IDR in a private clinic. Relative to the average income for Bantul residents (gross regional domestic product in 2013 was 12.1 million IDR per capita [12]), the service fee for the IHSP-Elderly program is affordable.

Demographic data for IHSP-Elderly members were extracted from IHSP-Elderly register books and interviews, then tabulated and descriptively analyzed. IHSP-Elderly members were interviewed until theme saturation was reached. Interviews were audio-recorded and transcribed in a local language (Javanese or Bahasa). Each transcript was analyzed by using thematic analysis via manual inductive coding. The first author (R.R.) translated the transcript from Javanese or Bahasa to English, and external translators, who were not involved in the study, verified the translation. The word use in the translation reflected the language capability of the participants. The coding framework was generated by the first author and subsequently reviewed and validated by the second author (B.B.) To ensure the validity of the analysis, the emergent themes were compared with the transcribed notes and verified by 5 participants (3 patients, 1 CHW, and 1 district health staff member) who volunteered to provide feedback.

## Results

Of the 15 participants, 11 were women; the mean age of the patients was 69.8 (standard deviation, 9.2) years (Table 2).

**Table 2:** Characteristics of Participants (N = 15), Study of the Role of a Community-Based Program for Elderly People with Hypertension, Indonesia, 2013

Characteristic	Members of IHSP-Elderly Program (n = 11)	Community Health Workers (n = 3)	District Health Staff (n = 1)
**Age, mean (SD), y**	69.8 (9.2)	55.7 (14.5)	41
**Women, n**	8	2	1
**Education**			
Below primary school	6	0	0
Primary school	2	0	0
High school	3	3	0
University	0	0	1
**Blood pressure, mean (SD), mm Hg**			
Systolic	165.5 (15.1)	NA	NA
Diastolic	94.5 (5.2)	NA	NA

Abbreviations: IHSP-Elderly, Integrated Health Service Post for the Elderly; NA, not available; SD, standard deviation.

### Field observation of a weekly meeting of the IHSP-Elderly program

The IHSP-Elderly meeting was attended by 25 members (18 women and 7 men). The 2-hour session was conducted in the afternoon in the front yard of a CHW’s house. The meeting began with a blood pressure check for each member, followed by group physical exercise. Most of the IHSP-Elderly members queued to get their blood pressure checked. The participants were jovial and appeared to have a good time; they shared jokes with CHWs and there was a lot of laughter. They made comments and suggestions freely each time the CHWs explained the blood pressure readings.

The physical exercise, featuring traditional music and simple verbal instructions, was facilitated by an instructor. The exercise was guided by appropriate imagery such as looking at the moon, water paddling, fist clenching, flying, and wave making. Most members followed the instructions with ease. A few had difficulty, but they tried to mimic the instructor’s gestures. Every now and then there was a ripple of their laughter. After the exercise finished, the CHWs provided light refreshments for all members.

### Thematic analysis of interviews

Three key themes emerged from the interviews: the central role of CHWs, multiple benefits for the elderly, and current limitations to providing optimal services.

#### Central role of CHWs in the community

The district health staff member emphasized the critical role of the IHSP-Elderly program in addressing hypertension in the community. She stressed that the program originated in the community to serve the community: “IHSP-Elderly plays a strategic role in our healthy aging program. It is a program by community and for community; as you know there are currently many elderly people. We can say that CHWs are health gatekeepers for the rural community.”

CHWs organized weekly physical exercise and monthly meetings, provided the venue for meetings, organized equipment and refreshments, planned materials and speakers for public education, and reported the IHSP-Elderly programs to the community health center. They seemed to be highly dedicated to their roles and to volunteering their time to serving the community in this way. Being unpaid volunteers, they were motivated by intangible benefits, such as the appreciation of community leaders, the trust of the community health center and members of IHSP-Elderly program, and the ability to contribute to the wellness and happiness of the community. “I love to share what I know with the elderly. Giving benefits to others makes me happy” (CHW).

I have been active as a CHW since 1997, with the trust of the community health center and village leader. They believe that I am still the best . . . despite my infirmity as I am getting older. The IHSP-Elderly members also ask me to remain [as a CHW coordinator]. For me personally, managing the IHSP-Elderly is lovely work.

In addition, the CHW prepared invitations and arranged home visits to increase participation of IHSP-Elderly members: “Elderly people tend to be forgetful. Giving invitations will remind them to come” (CHW). “In case there were inactive members, we would come to their house, greet them, and wish them all good health” (CHW).

CHWs also arranged public education programs on the physical, emotional, and spiritual aspects of healthy aging. These monthly programs featured both internal speakers (CHWs) and external speakers. The value of using CHW as speakers was their creativity in facilitating interactive discussions and encouraging elderly members to participate.

#### Multiple benefits for the elderly

**Being healthier.** The desire of older members to be healthier was the most motivating factor for participating in the IHSP-Elderly program. In particular, they perceived immediate health benefits from attending the physical exercise sessions, such as sleeping better and feeling more relaxed. “I feel better after the physical exercise. It’s quite easy for me. It makes me healthier” (Member). “It makes me healthier, relaxes my breathing, and I can sleep well after exercise. It is also helpful for my knee pain” (Member). “Members who feel healthier will be active in the IHSP-Elderly program” (CHW).

**Having peer support.** Peer support was an important motivator for participating in the IHSP-Elderly program: “I joined IHSP-Elderly a long time ago. It is hard for me now to follow physical exercise, getting older limits me from doing exercise, but CHWs motivated me just to come, and I enjoy the meet-and-greet with the peers” (Member). “One member, with a walker, always comes and joins IHSP-Elderly activities. She is glad to meet her peers and gets new information” (CHW). “The benefits come from the joyful meetings. They greet each other weekly. We can say that the active members are healthier than other elderly” (CHW).

**Accessing free health care.** Participation in the IHSP-Elderly program was also driven by the free access to health care. Members of IHSP-Elderly program were highly enthusiastic about having access to free blood pressure checks, regardless of who measured their blood pressure (the CHWs, health staff, or others). “Being examined is exciting, you know, as an elderly people, what a great opportunity we have to get an examination for free” (Member). “I am very glad. I always try to come if a blood-pressure check is announced” (Member). “I just put on the invitation that there will be blood pressure checks. They would be enthusiastic to come” (CHW). “They [CHWs] can do it, but sometimes it is done by CHC [community health center] staff. Three months ago there were medical students who also offered a blood-pressure checking program for free. That is okay, [whoever]” (Member).

#### Current limitations to providing optimal services

Although the members were enthusiastic about the CHW-led activities of the IHSP-Elderly program, they felt that there were some inconsistencies in what was offered; for instance, blood pressure checks were not done routinely. IHSP-Elderly meetings were conducted weekly, but only physical exercise could be offered weekly. The members suggested that a routine follow-up of blood pressure readings should also be arranged at least once a week. “It is not a routine program. If I am not mistaken, the last blood pressure check other than the one today was done last month, or two months ago. It would be lovely if they could make it weekly” (Member). “In some IHSP-Elderly, members can check their blood pressure. But as far as I know, not all of them have a capable CHW to do that” (District health staff member).

Additionally, the IHSP-Elderly program had only 1 properly functioning aneroid sphygmomanometer to measure blood pressure. This device was purchased in 2007 and had not been recalibrated in 6 years. There was no funding to purchase a new device. In this IHSP-Elderly program, only 1 CHW (female, 40 years old) was properly trained and had enough confidence to measure blood pressure; the other 2 CHWs did not feel as capable. “Actually I have been trained, but I felt nervous when people queued around me. I was shaking” (CHW). “She [the other CHW] did not come for a while because she fractured her hand. During that time, I could not take over her task [to measure blood pressure] as I am not skillful yet” (CHW).

CHWs recognized that their role in managing hypertension was restricted to early screening and monitoring, followed by referring patients to other health service providers. However, this study identified a gap between what elderly members wanted and what the IHSP-Elderly program could provide. Members preferred a simpler care pathway in which the CHWs could provide easier access to hypertension treatment: “I wish I could receive medication here [in IHSP-Elderly program] so that I needn’t go anywhere else” (Member).

Although the CHC provides a mobile health service for patient follow-up (including further examination and provision of medication), most IHSP-Elderly members did not use this service. Access to the mobile service was difficult; members had to travel approximately 3 kilometers to reach it. Furthermore, because hypertension is symptomless, some members were not motivated to travel. The following quotes illustrate the barriers to using the mobile health service:

When we identified hypertension cases, we would ask the patients to visit mobile health services, but most of them had difficulties accessing that service. I think it was related to transportation. It might also be because they did not feel any serious symptoms (CHW).

“I couldn’t go there. No one could take me over there” (Member). “I have told CHC staff that the members might not visit the mobile service, they need to ask their children or grandchildren to take them, and it is not easy in the working hours” (CHW).

## Discussion

This study highlights the potential benefits of involving CHWs in improving hypertension management in a developing country (Indonesia), supporting the findings of previous studies focusing on the role of CHWs in hypertension management (7,8,23). CHWs serve as a bridge between the community and the health care system, addressing community beliefs about diseases and supporting patients’ adherence to therapies, which is difficult to achieve in traditional health care settings (24,25). For example, nonpharmacological approaches play an important role in hypertension management, and CHW-based programs can offer exercise and health education activities. Some studies report that nonpharmacological approaches are not so well addressed as pharmacological approaches are in doctor–patient consultations (26,27). A CHW-based program may be well positioned to support hypertension management by providing information about lifestyle choices.

Self-monitoring blood pressure improves blood pressure control (28), but self-monitoring is difficult to facilitate in a low-resource population. Providing free blood pressure checks in a CHW-based program presents an opportunity to monitor patients between scheduled health service visits. Moreover, the patients’ enthusiasm for having their blood pressures checked in an IHSP-Elderly program is a good base on which to establish follow-up activities or to enhance self-management.

The limited capability of CHWs and a lack of resources (eg, calibrated sphygmomanometers) preclude a greater role for CHWs in providing regular blood pressure monitoring for IHSP-Elderly members. Research on the problems related to levels of infrastructure and CHW skill highlights the need for adequate training and supervision for CHWs (29–31). Training could be focused on how to 1) differentiate grades of hypertension, 2) help IHSP-Elderly members develop care plans for self-management, and 3) refer patients to other health care services. So that CHWs can provide accurate and up-to-date information to patients about medication for hypertension, training on the key messages about antihypertensive agents (their types, roles, and side effects) is needed, particularly in geographic areas with a high prevalence of hypertension and poor access to information. In such areas, demand for services from CHWs is high because of the constraints faced by the elderly in their ability to access existing follow-up services.

This study is the first of its kind from Indonesia to highlight the perspectives of patients, CHWs, and health policy advisors about a CHW-based program for hypertension management. Because of the methodology of qualitative studies and the fact that this study took place in 1 rural district and had a small sample, the results of this study cannot be deemed to represent the entire Indonesian population. However, because Bantul District is a typical rural district in Indonesia and the IHSP-Elderly program is a national program, our results support the likelihood that IHSP-Elderly program could play a larger role in CHW-based management of hypertension in other rural areas, even considering the barriers that may be encountered. Moreover, the findings of this study provide insights for the development of CHW-based programs in other settings with a similar social context.

This study highlighted how CHWs have the potential to liaise between rural communities and the wider health care system. The role of CHWs needs to be strengthened through targeted organizational support that aims to improve delivery of health care services and, when needed, referral to additional health care services. Further study is needed to identify the key factors for effective CHW-based programs in rural communities and the incorporation of these programs into the health care system.
